# Radiographic choice for skeletal maturation assessment: a systematic review of Cervical Vertebrae Method and Middle Phalanx Method

**DOI:** 10.1007/s11282-026-00912-y

**Published:** 2026-03-31

**Authors:** Angelo Michele Inchingolo, Grazia Marinelli, Francesca Calò, Claudia Ciocia, Antonio Rizzo, Francesco Inchingolo, Gianluca Martino Tartaglia, Massimo Del Fabbro, Andrea Palermo, Daniela Di Venere, Vito Crincoli, Alessio Danilo Inchingolo, Gianna Dipalma

**Affiliations:** 1 Interdisciplinary Department of Medicine, School of Medicine, University of Bari “Aldo Moro”, 70124 Bari, Italy; 2 Department of Biomedical, Surgical and Dental Sciences, Milan University, 20122 Milan, Italy; 3 Unit of Maxillo-Facial Surgery and Dentistry, IRCCS Foundation Ca’ Granda Ospedale Maggiore Policlinico, 20122 Milan, Italy; 4 Department of Experimental Medicine, University of Salento, 73100 Lecce, Italy

**Keywords:** Skeletal age, Cervical vertebral maturation, Pubertal growth spurt, Artificial Intelligence, Deep learning

## Abstract

**Objective:**

The aim of the present study was to conduct a systematic review of the literature to provide an updated guide for clinicians in choosing the most appropriate radiographic method for skeletal age assessment and timing the pubertal growth spurt (PGS).

**Methods:**

The review followed the PRISMA guidelines and used the PICOS strategy. Eligibility criteria focused on studies published in the last decade, directly comparing the Cervical Vertebrae Method (CVM) and the Middle Phalanx Method (MPM). Two reviewers performed the selection and a third reviewer intervened in cases of disagreement.

**Results:**

After screening, thirteen studies were included. The analysis revealed that CVM and MPM are highly correlated and equivalent biological indicators. From the perspective of precision and reliability, the MPM method demonstrated excellent intra-observer reliability and clearly revealed the PGS. Conversely, CVM method showed reproducibility that is not improved by increasing clinical experience, but it achieved excellent accuracy when supported by Deep Learning (DL) and Artificial Intelligence (AI) systems.

**Conclusion:**

This review revealed that although MPM remains an excellent biological indicator, the equivalence of results, combined with the routine adoption of cephalometric radiography and the increasing implementation of AI, solidifies CVM as the preferred radiographic method. Consequently, subjecting the patient to an additional hand-wrist radiograph is unwarranted, relegating MPM to the role of a reserve indicator.

**Clinical relevance:**

AI and DL technologies are expected to standardize and drastically improve the objectivity and accuracy of CVM assessments, representing a crucial shift toward objective clinical diagnostics to eliminate the acknowledged inter- and intra-observer variability and optimize radiation dose management. The protocol was registered with the Prospective International Register of Systematic Reviews under number ID 1,110,124.

## Introduction

Skeletal age assessment plays a crucial role in planning and timing therapeutic interventions in various medical fields [1–9]. In orthodontics, an accurate assessment of skeletal maturation is essential to maximize the effectiveness of treatments, particularly during active growth phases [10–20]. Accurate identification of the Pubertal Growth Spurt (PGS), for example, is essential for the application of orthopaedic appliances or for determining the optimal timing for orthognathic surgery [21–32]. Similarly, in forensic medicine, skeletal age assessment provides an essential objective parameter for complex legal and social decisions, such as identifying undocumented individuals [33–46].

Historically, several indicators of skeletal maturity have been used, including sexual maturation traits, chronological age, and dental and skeletal development [47–59]. Radiographic methods of the hand and wrist, such as those proposed by Greulich and Pyle (1959), Tanner-Whitehouse (1962), and Grave and Brown (1976), have been the “gold standard” for years [60–66]. These approaches relied on the study of mean ages of ossification of the carpal bones and phalanges [67–69]. Fishman (1982) and Björk both demonstrated a close correlation between skeletal maturation and hand development, linked maximum pubertal growth in height to specific stages of skeletal maturation of the hand and wrist [70–75]. However, while these methods have been extensively validated, they have a significant limitation: the need to expose the patient to additional radiation to acquire a specific hand radiograph [76–82]. In accordance with the ALARA (As Low As Reasonably Achievable) principle, research has therefore focused on alternatives that minimize radiological exposure, favoring the use of images already acquired for other diagnostic purposes [83–86].

### Cervical vertebral assessment method

The Cervical Vertebral Method (CVM) has established itself as a non-invasive diagnostic technique for assessing skeletal age in orthodontics. Its main advantage lies in the ability to use standard lateral cephalometric radiographs, already indispensable for cephalometric analysis, thus eliminating the need for additional specific radiographic exposures [87–95].

Lamparski in 1972 was among the first to propose the study of cervical vertebrae [96–100]. Subsequently, authors like Hassel and Farman (1995) and Paloma San Roman (2002) established important correlations with hand-wrist maturation [101–106]. In 2018, McNamara, Franchi and Baccetti proposed a revised classification of the CVM stages (CM1-CVM6), confirming the validity of the cervical vertebra as a biological indicator of skeletal somatic maturation and mandibular growth [107–111].

They developed a simpler and more easily applicable classification that includes six stages of skeletal maturation (CVM1-CVM6), based on the identification of specific changes in the shape of the vertebral body (rectangular, trapezoidal, square) and the concavity of the inferior margins of C2, C3, and C4:

- CVM1 (Onset): The inferior margins of C2, C3, and C4 are flat. C3 and C4 have a trapezoidal appearance. This indicates the prepubertal stage.
- CVM2 (Acceleration): A concavity appears at the inferior margin of C2. C3 and C4 remain trapezoidal. This coincides with the onset of the pubertal growth spurt.
- CVM3 (Maximum Acceleration): A noticeable concavity appears at the inferior margins of C2 and C3. C3 and C4 may begin to square off or remain trapezoidal. This stage is associated with the pubertal growth spurt.
- CVM4 (Deceleration): Concavity appears at C2, C3, and C4. C3 and C4 have a more square or horizontally rectangular appearance. This indicates a decelerated growth phase.
- CVM5 (Nearly Complete Maturation): C3 and C4 are decidedly rectangular (vertically), with persistent concavity in C2, C3, and C4.
- CVM6 (Complete Maturation): All vertebrae are square or vertically rectangular, with marked concavity. Indicates the end of skeletal growth.

**Fig. 1:**
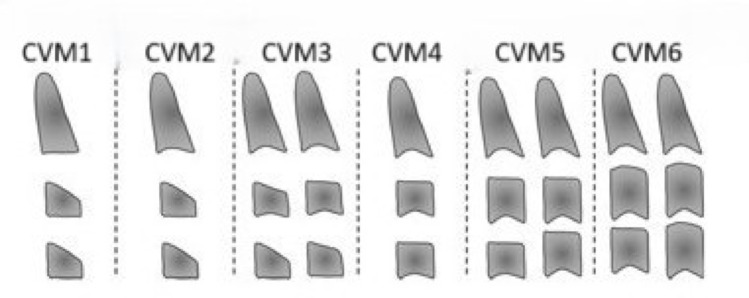
Stages of maturation of the cervical vertebrae proposed by Hassel and Farman

This classification is easy to interpret due to the association of well-defined geometric figures with the vertebral bodies and offers the possibility of analyzing the morphological characteristics of the cervical vertebrae even when the patient wears a thyroid protective collar [112–115]. However, the authors themselves acknowledge that staging can be imprecise due to gradual changes, sometimes making it necessary to consider intermediate stages [116–122]. Furthermore, the reliability of the method is proportional to the operator’s experience [123, 124]. Recent research, such as that of Singh et al. (2022) and Al-Maaitah et al. (2023), has further validated the accuracy of the CVM, confirming its reliability in predicting peak mandibular growth [125–128]. O’Reilly and Yaniello also confirmed the correlation between vertebral and mandibular maturation with the different growth stages during puberty [129–131]. Other classification schemes, such as that of Mito et al. (2002), although presenting slight variations, are based on similar principles of morphological evaluation [132, 133] (Fig. 1).

### The Middle Phalanx Method

The Middle Phalanx Method (MPM) focuses on the analysis of specific bone changes observed in the third phalanx of the middle finger [134–136]. Ossification and fusion of the epiphyses in this region represent key events closely related to the pubertal growth spurt [137–139]. Several classifications, including those by Hagg and Taranger (1980) and Fishman (1982), describe the stages of skeletal maturation of the third phalanx of the middle finger through a sequence of events based on the opening and closing of the growth plate and changes in the shape of the epiphysis [140–142].

Typical stages include:

Stage MPM1 (Prepubertal): The epiphysis is narrow, like a capsule, smaller than the diaphysis. The growth plate is wide and open.

Stage MPM2 (Beginning of Spurt): The epiphysis and diaphysis are similar in width. The growth plate begins to narrow.

Stage MPM3 (Growth Surge): The epiphysis takes on a “hood” shape, extending beyond the diaphysis. The growth plate is still clearly visible but narrower. This stage is often associated with the pubertal growth spurt.

Stage MPM4 (Deceleration): The epiphysis begins to fuse with the diaphysis in some places. The growth plate is closing.

Stage MPM5 (Nearly Complete Fusion): Most of the growth plate is closed, with only small remnants visible.

Stage MPM6 (Complete Maturation): The growth plate is completely closed, with epiphysis-diaphysis fusion complete.

**Fig. 2:**
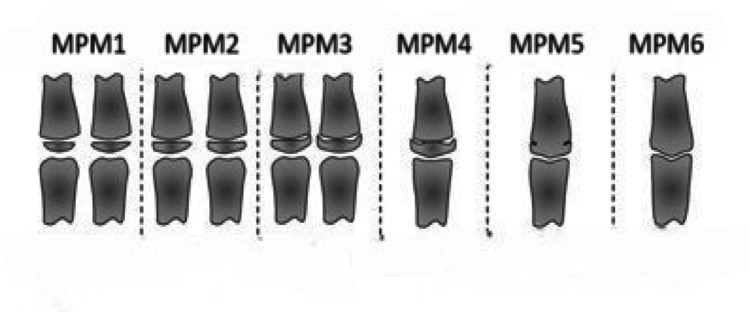
Stages of maturation of the middle phalanx of the third finger (MPM) proposed by Fishman, Hagg, and Taranger

MPM is another well-established technique, often preferred due to its high correlation with the PGS and the specificity of its maturation markers [143, 144] (Fig. 2).

### New perspectives and future directions

In recent years, the introduction of quantitative techniques based on Deep Learning (DL) and the application of Artificial Intelligence (AI) algorithms have expanded the diagnostic potential of CVM and MPM [145–149]. Recent studies (Makaremi et al., 2019; Kavousinejad et al., 2024; Jiang et al., 2025; Mohammad-Rahimi et al., 2022) have demonstrated how AI can improve the objectivity of CVM assessments, reducing interobserver variability and improving accuracy and repeatability. These developments represent an important step forward in the standardization of skeletal assessment [150–152].

This systematic review aims to analyze and compare the methods based on the analysis of the cervical vertebrae and the third digit phalanx, with particular attention to the efficacy, reliability, limitations and technological updates of each approach [153–156]. The aim is to provide an up-to-date and critical overview for clinicians and researchers, facilitating the selection of the most suitable radiographics method based on specific diagnostic needs and recent scientific findings for the assessment of peak growth and skeletal maturation [157, 158].

## Materials and methods

This systematic review followed the Preferred Reporting Items for Systematic Reviews and Meta-Analyses (PRISMA) and was registered in the International Prospective Register of Systematic Reviews (PROSPERO), under registration number ID 1,110,124.

### Eligibility criteria

The PICOS acronym was used to define the eligibility criteria based on the following research question formulated for this systematic review: “What is the most appropriate method for assessment of skeletal age in growing patient?”

The following process was used to construct the PICOS model:

- (P) Population: Growing patients.
- (I) Intervention: Tele-rx and phalanx rx.
- (C) Comparison: CVM method and MPM method.
- (O) Outcome: Accuracy in predicting the pubertal growth spurt (PGS), correlation between CVM and MPM stages, and inter- and intra-observer reproducibility (reliability) of both methods.
- (S) Study design: Cross-sectional, retrospective or clinical studies.

### Inclusion criteria

This review included observational cross-sectional, retrospective or clinical studies involving growing patients (< 18 years) without any syndrome. No studies restrictions were imposed with regards to sex or ethnicity. Three reviewers evaluated all relevant papers based on the following chosen criteria:

1. human subject studies;
2. full-text articles with open access and written in English;
3. scientific studies evaluating the use of CVM and MPM methods;
4. studies that were published in the last 10 years.

### Exclusion criteria

1. Articles written in other languages than English,
2. off-topic studies, reviews, letters to the authors or comments, ineligible study designs, ineligible outcome measures, ineligible populations, reviews, in vitro and animal studies.

### Search strategy

We limited our search to English-language papers published between January 1, 2015, and May 7, 2025, in PubMed, Scopus and Web of Science that were relevant to our topic. In the search approach, the Boolean keywords (“Skeletal Growth Evaluation”) AND (“Cervical Vertebrae” OR “Middle Phalanx”) were used. We selected these phrases because they most accurately reflected our investigation’s aim, which was to compare the diagnostic value of cervical vertebrae maturation and third phalanx maturation in assessing skeletal age.

### Study selection

Articles were analyzed in two phases. In phase 1, two reviewers (F.I and A.P.) separately reviewed the titles and abstracts of all references. Articles that did not meet the eligibility criteria were excluded. In phase 2, the same reviewers independently read the full texts of the preselected articles. Divergences of opinion between the two reviewers were discussed. If no consensus was reached, a third reviewer (A.M.I) intervened to deliver the tie-breaking vote. The reviewers were calibrated to ensure a greater level of agreement. The studies were independently evaluated by the reviewers using a special electronic form designed according to the following categories: authors, year of study, aim of the study, materials and methods, and results.

### Data extraction

Two reviewers (F.I and A.M.I) extracted data from the studies included. Discrepancies were resolved through dialog with a third reviewer. The following data were collected: study characteristics (authors, year of publication, country, and study design), characteristics of the clinical assessment, details of the results, and conclusions. When data were incomplete, the article was excluded.

### Quality assessment

Two reviewers, F.I and A.M.I., evaluated the included papers’ quality using the ROBINS-I tool (Cochrane Bias Methods Group and the Cochrane Non-Randomised Studies of Interventions Methods Group Creative Commons Attribution Non Commercial No Derivatives 4.0 International License). In order to evaluate the possibility of bias in the outcomes of non-randomized trials comparing the health impacts of two or more therapies, ROBINS-I was created. Each of the seven evaluated points was given a bias degree. F.I., the third reviewer, was consulted in the case of disagreement until a consensus was reached. The reviewers were instructed on how to use the ROBINS-I tool and adhered to the guidelines in order to assess the potential for bias in seven different domains: confounding, participant selection, intervention classification, deviations from intended interventions, missing data, outcome measurement, and choice of re-ported results. Discussion and consensus were used to settle any differences or conflicts amongst reviewers in order to improve the assessments’ objectivity and uniformity. In situations when agreement could not be reached, the final decision was made by a third reviewer. An extensive assessment of potential biases in the non-randomized studies included in this study was made possible by the use of ROBINS-E for bias assessment. This contributed to the overall evaluation of the calibre and dependability of the results by pointing out the evidence base’s advantages and disadvantages. The writers of this review were able to reach more informed interpretations and conclusions based on the facts at hand by taking the risk of bias into account.

## Results

### Study selection and characteristics

**Fig. 3:**
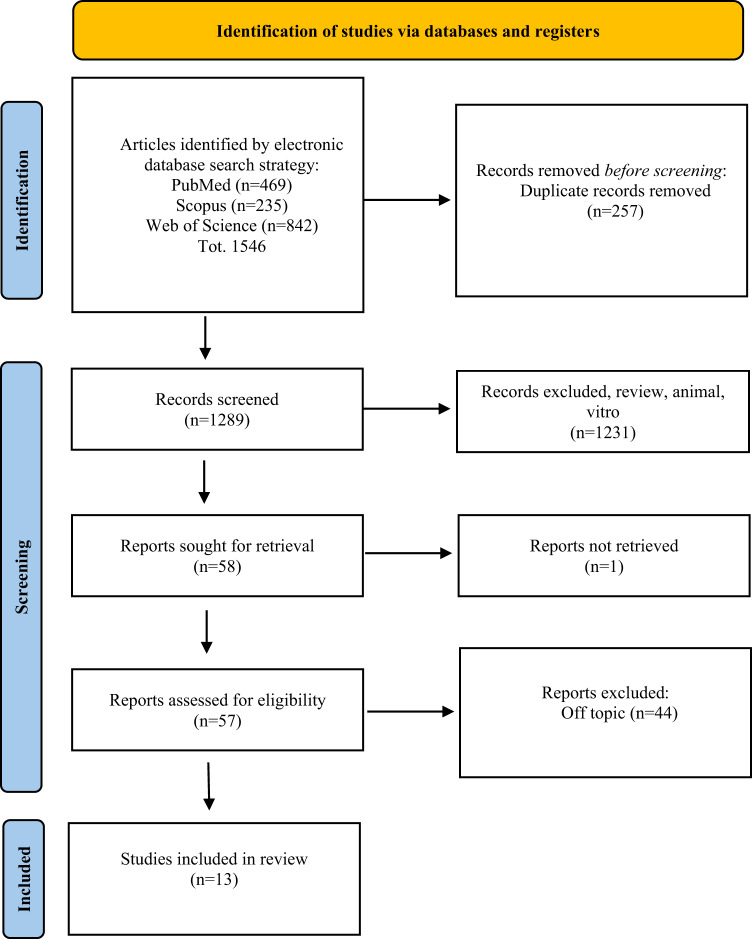
PRISMA flow diagram

Figure 3 shows the flow diagram of a systematic review carried out using the Preferred Reporting Items for Systematic Reviews and Meta-Analyses (PRISMA) reporting criteria. The diagram describes the search strategy, inclusion, and exclusion of publications at each stage of detection.

A total of 1546 papers were identified in three databases, including PubMed (469), Web of Science (842), and Scopus (235). After screening, 257 duplicated articles were removed. Following the exclusion of in vivo/invitro studies, animal studies and systematic reviews, 57 records were assessed for eligibility by analyzing the title and abstract. After full-text eligibility, 13 studies were included in the finale analysis. The process is summarized in Fig. 3. The other characteristics of the studies are shown in Table 1.

**Table 1:** Analysis of the study included in the discussion section

Authors	Study design	Sample size	Aim of the study	Results	Conclusions
Alhamady A.M et al., 2024 [159]	Retrospective cross-sectional study	647 Images	The study evaluated the reliability of an objective staging system based on measurements for CVM.	Intra-observer reliability was very high (0.948) and inter-observer reliability was also very high (0.967). The Superior Wall Inclination Angle of C3 and C4 vertebrae was significantly larger in the post-pubertal group compared to the pre-pubertal and pubertal groups (*P* < 0.001). The objective method showed a strong correlation with chronological age.	The Superior Wall Inclination Angle proved to be a reliable indicator of skeletal maturation. The objective CVM staging system, incorporating measurements such as Superior Wall Inclination Angle, concavity depth and body shape can be used for a quantitative assessment of the skeletal maturation level.
Bulut M. et al., 2024 [160]	Clinical Study	1000 Individuals	The study compared the relationship between HWM and CVM stages with chronological age in 1000 patients.	The correlation between CVM and HWM, without gender or malocclusion differentiation, was found to be very high with a Spearman coefficient of 0.887. The correlation remained strong even when differentiated by gender and malocclusion class.	Given the strong correlation, the use of hand-wrist radiographs alone (which expose the patient to an additional radiation dose) is not necessary for orthodontic treatment planning, as the CVM method, which utilizes the routinely performed lateral cephalometric radiograph, is sufficient
Eninanç I. et al., 2023 [161]	Retrospective Study	144 Individuals (6-17years)	The study analyzed 144 subjects compares FD measurements in different regions (radius, phalanges, cervical vertebrae C2, C3, C4) with HWM stages and CVM stages	A very strong positive correlation was found between HWM stage and CVM stage. FD measurements on the radius showed moderate positive correlations with both HWM and CVM stage. Radius FD values also correlated positively with C2, C3, C4 vertebrae and phalanx FD values, as well as chronological age.	FD measurements performed on hand-wrist radiographs can provide useful information for skeletal maturation stage assessment. Specifically, FD measurements obtained from the radius are considered important and more reliable for predicting the skeletal maturation stage.
Hayat S. et al., 2024 [162]	Comparative cross- sectional study	110 Individuals 55 M 55 F	To compare CVM and MPM for assessment of Pubertal Growth Spurt	A significant association and a strong correlation (0.937) were found between CVM and MPM stages (*P* < 0.001). A very good Kappa correlation value of 0.82 was reported between CVM and MPM stages.	A significant strong correlation exists between CVM stages and MPM maturation stages. Females exhibit signs of earlier physiological maturity.
Jiang F. et al., 2025 [163]	Clinical study	2100 Images	The study developed a DL based system called CVnet for QCVM.	CVnet system alone demonstrated high accuracy and excellent reliability in quantitative CVM staging. CVnet significantly improved the diagnostic accuracy of six junior orthodontists from 83.65% to 92.4%. Results were statistically similar to those of a senior orthodontist.	CVnet system enables precise landmark localization and accurate maturation staging. CVnet is a valuable tool for orthodontists, particularly in improving diagnostic consistency.
Kavousinejad S. et al., 2024 [164]	Clinical case	980 Images	The study aimed to develop a semi-automated approach using ML based on cervical vertebral dimensions.	The proposed model achieved an accuracy of 99.49%. The model utilized feature engineering, simplified landmark selection and an auto error reduction.	By leveraging feature engineering, simplified landmark selection and data augmentation, a model was developed for the accurate assessment of skeletal maturation for clinical applications
Khadilkar V. et al., 2024 [165]	Clinical case	493 Images (226 F)	The study developed a simplified method for bone age estimation (S-Ba) using only three bones of the hand and wrist.	S-Ba method showed a strong correlation with both the Greulich and Pyle method and Tanner-Whitehouse 3 methods	Due to its simplicity and high correlation with the Tanner-Whitehouse 3 methods, it is considered ideal for clinical use.
Makaremi et M. et al., 2019 [166]	Original article	600 Images	DL and Convolutional Neural Network system successfully performed automatic classification of CVM stages. The highest accuracies were achieved for the extreme maturation stages (CVS1 and CVS6)	The average classification accuracy on a test set of 120 images was 80%. The highest accuracies were obtained for the extreme maturation stages (CVS1 and CVS6), reaching 93.2% for CVS1 and 93.9% for CVS6.	The application of DL and AI tools demonstrated success in CVM stage determination. Automation of the CVM process leads to time savings, increased efficiency and repeatability.
Meghana H.C et al., 2016 [167]	Clinical study	112 Individuals	The study compared skeletal maturation methods based on MPM and CVM.	A very significant correlation was found between MPM and CVM stages (*P* < 0.001). The MPM method showed excellent intra-observer reliability with a Kappa coefficient (κ) of 0.89. PGS was observed at the transition between Stage 3 and Stage 4 of the middle phalanx, corresponding to the transition between Stage 2 and Stage 3 of the cervical vertebrae.	The results confirmed a highly significant correlation between the maturation stages of MPM and those of the CVM. The MPM method is a reliable and accurate indicator for skeletal maturity assessment. It can be used as a sole method for assessing skeletal maturity, especially in orthodontics, as an alternative to CVM method.
Mirabelli L. et al., 2023 [168]	Retrospective Study	98 Individuals	Comparison between two methods of Skeletal Growth Evaluation: CVM and MPM method.	88.8% of patients showed complete agreement between the MPM and CVM methods. A high degree of statistical correlation was found between the two methods.	MPM and CVM methods show satisfactory agreement. MPM is a valid and alternative indicator to CVM for identifying the pubertal growth spurt. MPM is reliable and easy to interpret, allowing monitoring without an additional lateral cephalometric rx
Mohammad-Rahimi H. et al., 2022 [169]	Pilot study	890 Images	The study proposed CNN for the automatic classification of CVM stages and the prediction of PGS.	The model achieved a CVM stage classification accuracy of 94.6% on the test set. The model demonstrated a high capability to predict the presence or absence of the PGS, with an accuracy of 94.3% on the test set. The Cohen’s Kappa coefficient was 0.89 (almost perfect agreement), indicating that the automatic classification is highly reliable.	The DL-based system can be used as a fast, accurate, and reliable tool for classifying the cervical maturation degree and for predicting PGS. Such an AI system has the potential to assist orthodontists in identifying the optimal treatment timing.
Pamukcu U. et al., 2022 [170]	Clinical study	120 Images	The study analyzed the correlation between FD values of the cervical vertebrae (C2, C3, C4) and skeletal maturation stages determined by HWM method and CVM method.	FD values of cervical vertebrae (C2, C3, C4) showed a moderate positive correlation with both HWM method and the CVM method. FD values of C3 and C4 showed a slightly stronger correlation compared to C2.	The FD values of the C2, C3, and C4 cervical vertebrae show compatibility with the skeletal maturation stages determined by the CVM and HWM methods. FA proved to be a promising method to support the quantitative assessment of skeletal maturation.
Rongo R. et al., 2015 [171]	Clinical case	50 Images	The study evaluated the reproducibility inter-observer and intra-observer agreement of the CVM method by comparing three groups with different levels of orthodontic experience: Junior < 1 year, Postgraduate 2–4 years, Specialists ≥ 7 years.	The Junior group achieved the highest inter-observer agreement coefficient (Kendall’s W up to 0.87), indicating near-perfect agreement. The Specialist group obtained the lowest W values (down to 0.61). The Junior group also showed the highest intra-observer perfect agreement percentage (57.8%).	The reproducibility of the CVM method is not improved by the level of orthodontic experience. The group with the lowest level of orthodontic experience had the best performance across all investigated parameters.

### Risk of bias

The risk of bias across the included studies has been systematically evaluated and summarized in Table 2. The quality assessment was based on seven key domains: con-founding bias, measurement of exposure, participant selection, post-exposure interventions, missing data, measurement of outcomes, and selection of reported results. These domains were used to evaluate potential threats to the internal validity of each study. Overall, the majority of the included studies demonstrated a moderate risk of bias, with some methodological concerns.

### Summary of results

A strong and significant correlation was found between the skeletal maturation stages determined by the CVM and MPM methods. Direct comparative studies report Spearman’s rank correlation coefficients ranging from 0.887 to 0.972, with some studies reporting an excellent Kappa agreement coefficient, reaching 0.82. This high concordance confirms that both methods are equivalent indicators of the pubertal growth timing.

From the perspective of precision and reliability, the MPM method demonstrated excellent intra-observer reliability (κ = 0.89) and clearly revealed the PGS. Similarly, the CVM, although having shown a reproducibility that does not improve with increasing clinical experience, has been validated by objective measurement-based systems and achieved excellent accuracy (up to 99.49%) when supported by DL and AI systems. Such AI systems not only automate CVM staging with high accuracy (95.2%) but also significantly improve the diagnostic performance of junior orthodontists. Furthermore, FD provided quantitative support, showing a moderate but significant correlation between the FD of the C3/C4 vertebrae and the CVM/MPM stages. Consistent across all studies, an earlier skeletal maturation in females compared to males was confirmed.

**Table 2:**
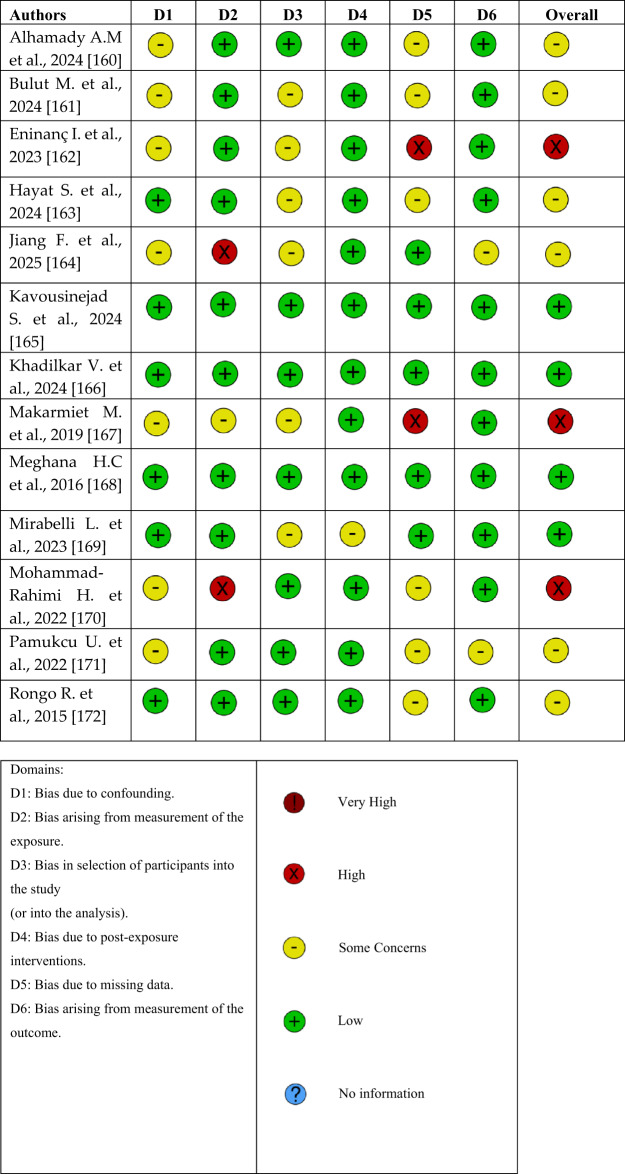
The quality assessment and Risk of Bias of included studies

## Discussion

Assessing skeletal maturity is a cornerstone of orthodontic and orthopaedics treatment planning in growing patients [172]. Specifically, the studies included in this review consistently showed that CVM and MPM are highly correlated, with correlation coefficients ranging from 0.887 to 0.972, confirming their biological equivalence. Chronological age, being an unreliable indicator of individual bone development, has necessitated the adoption of alternative methods to identify the biologically most appropriate time for intervention, particularly the pubertal growth spurt [173]. The systematic evaluation of the selected studies confirms a strong biological correlation between cervical vertebrae and middle phalanx maturation stages, with correlation coefficients consistently ranging from 0.887 to 0.972. Some authors argue that although hand and wrist radiography (HWM) is a well-established and reliable method for determining skeletal age and growth spurt, the need for additional radiography and the resulting radiation exposure has led to the establishment of other methodologies, most notably cervical vertebrae assessment (CVM) and the (MPM). Regarding clinical application, the MPM demonstrated high reliability, particularly in the age range of 8 to 12 years, with a reported intra-observer agreement of κ = 0.89. However, its main disadvantage is the requirement for an additional X-ray (hand-wrist or isolated middle phalanx), which increases the radiation dose for the patient. On the other hand, the Cervical Vertebrae Maturation (CVM) method offers a significant clinical advantage as it utilizes the lateral cephalogram, a standard diagnostic tool already required for orthodontic planning. This aligns with the ALARA (As Low As Reasonably Achievable) principle by avoiding unnecessary radiation. Furthermore, the integration of Artificial Intelligence (AI) in CVM analysis is addressing previous concerns regarding inter-observer subjectivity, providing automated, highly reproducible assessments that enhance diagnostic precision in modern clinical settings [174].

### The Cervical Vertebrae Method (CVM)

The CVM method, which analyzes cervical vertebrae on routinely performed cranial cephalometric radiographs, offers the primary advantage of avoiding additional radiation exposure to the patient, complying with the ALARA (As Low As Reasonably Achievable) principle. This makes it a radioprotective and effective option [169].

Despite its well-defined criteria, this method can be subjective, and reproducibility can vary with operator experience. The study by Roberto Rongo et al. highlighted that the reproducibility of the CVM method is not directly proportional to clinical experience [171]. Conversely, clinicians with recent and specific academic training in the method demonstrated higher intra- and inter-observer agreement than their more experienced colleagues who were not adequately trained in CVM [171]. This underscores the crucial importance of targeted training to ensure reliability [170, 171]. Indeed, even for specialists with more than 7 years of experience, the percentage of perfect interobserver agreement ranged between 42.3% and 46.3%, while less experienced groups showed higher agreement [164]. The accuracy of the CVM can also be compromised by overlapping anatomical structures, artifacts, or poor radiographic quality, making it difficult to accurately identify vertebral morphologies [163, 166].

The CVM has proven to be particularly effective in prepubertal and pubertal age, but its discriminatory ability declines in the advanced postpubertal stages, when vertebral changes are less significant and growth has almost ceased [162, 165]. The study by Luca Mirabelli et al. suggests that the CVM is less precise in identifying the exact moment of growth cessation [168]. The presence of congenital or acquired anomalies of the cervical vertebrae can also render the method inapplicable or lead to misinterpretations [167, 168].

To reduce subjectivity and improve reliability, Alhamady et al. proposed an objective CVM staging system, which showed high intra-observer (0.948) and interobserver (0.967) reliability when there was initial agreement between examiners [159]. The same study proposes a simplified and objective CVM staging system, grouping the six traditional stages into three main phases (prepubertal, pubertal, post-pubertal) through linear and angular measurements [159]. This approach also makes staging more suitable for artificial intelligence (AI) tools and automated applications, suggesting a future evolution of skeletal diagnosis towards automation [159, 166].

Research is indeed moving toward the use of software and AI to automate and standardize assessment, reducing reliance on the human eye and improving reproducibility, as reported by numerous studies such as Shahab Kavousinejad et al., Masrour Makaremi et al., Fulin Jiang et al., and H. Mohammad-Rahimia et al. AI is revolutionizing CVM staging, aiming to eliminate subjectivity and reduce analysis time [163, 164, 166, 169]. The CVnet system, based on DL, showed an average localization error of cervical vertebrae landmarks of 0.66 ± 0.46 mm and a diagnostic accuracy of 95.2% for QCVM staging and significantly improved the diagnostic accuracy of junior orthodontists from 83.65% to 92.4% demonstrating the potential for more objective assessment [163, 164, 166, 169].

Recent studies have explored the use of fractal analysis (FA) to assess trabecular bone architecture [170]. However, the study by Umut Pamukcu et al. showed no significant correlation between fractal values of cervical vertebrae (C2, C3, C4) and chronological ages or HWM and CVM stages, highlighting the limitations of applying this technique specifically to the vertebrae [170]. Only at C4 was a modest negative correlation observed with HWM stages in females [170].

###  The Middle Phalanx Method (MPM)

The (MPM) is another well-established and widely studied technique for assessing skeletal age, focusing on the analysis of specific bone changes observed in the third phalanx of the middle finger, as advocated by Meghana H.C [167]. This method is often preferred due to its high correlation with the pubertal growth spurt and the specificity of its maturation markers [167]. Stages in which the epiphysis completely covers the diaphysis or begins to fuse typically correlate with the pubertal growth spurt, offering a narrower and perhaps more precise window for identifying the growth spurt than the CVM [162, 170].

However, as Musa Bulut and Luca Mirabelli point out, the main limitation of MPM is the need for a specific X-ray of the phalanx [160, 168]. This implies additional patient exposure to ionizing radiation, even if the dose is very low [160]. This practice violates the ALARA principle unless strictly justified by a clinical indication not met by other available X-rays. Finger X-rays may not always be justified or available in all clinics or medical facilities [160, 168].

Although considered good, some authors argues that even in MPM, there can be some subjectivity in interpreting the phases, especially in the transitional phases, requiring adequate operator training and calibration. Accuracy may decrease in the very early (infancy) or very late (late adulthood) phases, when bone changes are less dynamic or have completely ceased. The reproducibility of MPM is generally considered high, both intra- and inter-operator, thanks to maturation phases based on more objective changes in the growth plate and epiphyseal shape, which are less susceptible to subjective interpretations than vertebral shapes [160, 168].

The MPM, introduced by Perinetti, represents a good compromise, offering a detailed assessment with low radiological impact, as argued by Meghana Abdel-Kader et al. supported the validity of the MPM method, emphasizing its ability to provide consistent results across different observers and at different time points [167]. AI-based solutions are also being explored for the MPM to automate classification and ensure even greater consistency [166, 167].

Meghana HC, Sumbal Hayat, and Luca Mirabelli showed that skeletal maturation of MPM and cervical vertebrae progresses with advancing chronological age [162, 167, 168]. A 2024 study by Musa Bulut compared the relationship between hand-wrist and cervical vertebrae maturation with chronological age, finding a correlation coefficient of 0.887 when sex and malocclusion type were not differentiated [160]. This suggests that hand-wrist radiography may no longer be strictly necessary if a latero-lateral teleradiography is already available, given the high correlation between the two methods [160]. A 2019 study by Sumbal Hayat compared the CVM and MPM methods for assessing pubertal growth spurt, finding a significant association between the two methods [162]. Females tended to reach maturation earlier than males in both methods [162].This authors observed that the MPM method is highly correlated with the CVM (*r* = 0.97), although they reported a slight decrease in concordance in the stages following puberty [162]. However, in the age group between 8 and 12 years, the MPM proved to be particularly useful and reliable [162].

HC Meghana et al. confirmed that MPM stages show a highly significant correlation with CVM stages, demonstrating that both methods can be used interchangeably to estimate PGS [167]. Vaman Khadilkar et al. introduced a simplified method based on the observation of only three bones (radius, ulna, and middle phalanx) for bone age estimation, facilitating clinical application without compromising diagnostic reliability [165]. This method showed a strong positive correlation with standard methods (Greulich and Pyle, Tanner-Whitehouse) and no significant interobserver variability, suggesting greater clinical applicability than more complex methods [165].

The correlation between Fractal Dimension (FD) values of the cervical vertebrae (C2, C3, C4) and the HWM maturation and CVM methods was also examined [170]. Significant positive correlations were found between the FD values of the radius and HWM/CVM (*r* = 0.559, *P* = 0.001; *r* = 0.528, *P* = 0.001, respectively) [165, 170]. This indicates that FD based methods could offer another comparative perspective [165]. İlknur Eninanç et al. demonstrated that FA applied to the radius and phalanx can offer a reliable quantitative measurement of bone development, finding a good correlation with CVM stages [161].

An often overlooked aspect concerns the influence of the type of malocclusion on skeletal maturation [161]. The study by Musa Bulut et al. showed that females with Class II malocclusion had higher mean ages in the advanced stages of CVM and HWM compared to peers with other Classes, while no significant differences were observed in males [160]. This result highlights the need to personalize the diagnosis based on the type of skeletal anomaly and not only on sex or age [160, 161].

### Clinical implications and future research

The integration of artificial intelligence in orthodontics is opening new frontiers for assessing skeletal maturity [168]. DL and machine learning (ML) algorithms can locate landmarks and classify maturation stages with an accuracy comparable to or superior to that of less experienced orthodontists [160, 168]. This reduces the dependence on clinical experience and manual analysis time [160].

Shahabi et al.‘s study achieved excellent results using a DL algorithm to identify CVM stages on cephalometric radiographs, with accuracies exceeding 80% [165]. Similarly, Kavousinejad et al. combined geometric measurements, achieving an accuracy exceeding 99%, demonstrating that AI-based automation can overcome subjective variability of the human observer [164]. A significant contribution also comes from Fulin Jiang et al.‘s study, which developed CVnet, a fully automated DL system for QCVM staging [163]. Unlike many previous approaches, CVnet quantitatively analyze all six stages (CS1–CS6) using dynamic localization of anatomical landmarks, with robust and clinically applicable results [163]. The model was tested on 2100 multicenter images, demonstrating a 10.24% improvement in diagnostic accuracy by junior orthodontists and a reduction in analysis time of over 6 min per image [163, 164]. However, the study acknowledges limitations in CVM discrimination, as well as the lack of hand-wrist radiographs and chronological age data, factors that will be integrated into future versions of the system for more comprehensive skeletal analysis [164].

These systems promise to dramatically reduce analysis time and eliminate inter-operator variability, providing immediate and objective diagnoses [163, 164]. Current challenges include the need for large and diverse training datasets and validation in real-world clinical settings [163].

 [163, 165]. Hand-wrist or single-phalangeal radiography could be reserved for specific cases or when an adequate cephalogram is not available [163].

Future research should focus on large-scale validation of AI algorithms in various clinical settings [163, 165]. It will be crucial to integrate these tools into daily clinical practice and develop standardized guidelines for their use [163, 164, 168].

Despite the body of research, more longitudinal studies following the same group of individuals over time are still needed to fully validate the long-term predictive accuracy of the CVM and MPM methods and to better understand individual variations in growth [160, 169]. The long-term goal is precision medicine, where skeletal age assessment is personalized based on the patient’s individual genetic, ethnic, and clinical characteristics [163]. Genomics and proteomics may one day provide predictive biomarkers that complement or surpass current radiographic techniques [163, 172]. A quantitative synthesis (meta-analysis) of the correlation coefficients was considered during the study design phase. However, a formal meta-analysis was not performed due to the high heterogeneity observed among the included studies, specifically regarding population ethnicities, chronological age ranges, and variations in the statistical methodologies used to calculate correlation. According to PRISMA guidelines, such diversity in study designs could lead to biased or misleading pooled estimates; therefore, a qualitative systematic synthesis was deemed more appropriate for this review.

The future may not lie in the exclusive use of a single method, but in integrating multiple indicators to obtain a more robust and personalized estimate of skeletal age [164]. This will provide more accurate, reproducible, and efficient diagnoses, with a constant focus on minimizing radiation exposure [163]. Combining data from CVM and MPM, perhaps weighting their reliability based on the patient’s growth stage, could provide a more comprehensive assessment [169, 172]. For example, CVM could be used as an initial screening test, and MPM as a confirmatory test in cases where greater precision in the growth spurt is required [163]. Research is exploring the correlation between radiographic growth indicators and biological or hormonal markers (e.g., growth hormone levels, IGF-1, dental maturation) [166, 168]. Although direct integration in the clinic may be complex, these studies contribute to a deeper understanding of growth processes [170, 171]. Integrating radiological data with anthropometric (height, weight) and medical history (chronological age, pubertal status) data can further refine the estimate, creating more sophisticated predictive models [163, 172]. The advent of digital radiology has revolutionized image acquisition and archiving, providing the basis for the application of advanced algorithms [167]. AI, particularly ML and DL, is emerging as the most promising frontier for overcoming the limitations of subjectivity and variability of traditional methods [163, 168, 170, 172].

## Conclusions

The choice of the most appropriate method for skeletal maturation assessment depends on the clinical context, diagnostic objectives, and a careful consideration of radiological exposure. Our findings demonstrate that the Cervical Vertebral Maturation (CVM) and Middle Phalanx Method (MPM) are highly correlated biological indicators for identifying the pubertal growth spurt, with correlation coefficients ranging from 0.887 to 0.972.

While both methods provide equivalent biological information, the CVM method offers significant clinical practicality in orthodontics. Since lateral cephalometric radiographs are already a standard component of the orthodontic diagnostic records, the CVM method allows for skeletal assessment without additional radiological exposure, strictly adhering to the ALARA (As Low As Reasonably Achievable) principle. Although some inter-operator variability has been noted, the integration of Artificial Intelligence (AI) is currently enhancing the accuracy and objectivity of this method.

On the other hand, the MPM remains a highly reliable and valid alternative, particularly when cephalometric images are unavailable or when a specific assessment of the hand-wrist area is clinically indicated. However, given the high correlation between the two methods, the routine addition of hand-wrist radiographs may not be justified when a cephalogram is already available.

In summary, the future of skeletal age assessment is moving towards intelligent automation and personalized diagnostics. Rather than establishing the absolute superiority of one method, this review emphasizes that the CVM method stands out for its clinical efficiency and its role in minimizing radiation exposure within a standard orthodontic workflow.

## Data Availability

The data presented in this study are available on request from the corresponding author. The data are not publicly available due to privacy and ethical restrictions.

## Abbreviations

AI: Artificial Intelligence
ALARA: As Low As Reasonably Achievable
CNN: Convolutional Neural Network
CVM: Cervical Vertebral Maturation
DL: Deep Learning
FA: Fractal Analysis
FD: Fractal Dimension
HWM: Hand-Wrist Method
ML: Machine Learning
MPM: Middle Phalanx Method
OPT: Orthopantomography
PGS: Pubertal Growth Spurt
QCVM: Quantitative Cervical Vertebral Maturation
